# Milkweed Fiber Nonwovens for Sustainable Thermal and Acoustic Building Insulation

**DOI:** 10.3390/ma18163821

**Published:** 2025-08-14

**Authors:** Deborah Lupescu, Mathieu Robert, Said Elkoun

**Affiliations:** 1 Department of Civil Engineering, Université de Sherbrooke, 2500 Boulevard de l’Université, Sherbrooke, QC J1K 2R1, Canada; 2 Department of Mechanical Engineering, Université de Sherbrooke, 2500 Boulevard de l’Université, Sherbrooke, QC J1K 2R1, Canada; said.elkoun@usherbrooke.ca

**Keywords:** milkweed hollow fiber, air-laid nonwoven, acoustic, thermal properties

## Abstract

This study investigates the use of a local fiber, specifically milkweed that grows in Quebec, Canada, for nonwoven building applications. Milkweed is a natural fiber with an ultra-lightweight hollow structure that provides excellent acoustic and thermal insulation properties. To provide three-dimensional stability to nonwovens, milkweed fibers were blended with a low-melt fiber composed of a polyethylene terephthalate core and a polyolefin sheath (LM 2.2), and polylactic acid (PLA) fibers. Several nonwovens with different fiber contents were manufactured using an air-laid Spike process. The nonwovens were compared with a commercially available thermal insulation material made of 100% hemp. The thermal conductivity and thermal resistance were measured at different temperatures. The sound absorption coefficient of the nonwovens was determined both using an impedance tube and the Johnson–Champoux–Allard (JCA) acoustic model. The results showed that all nonwovens exhibit thermal conductivity values below 70 mW/m·K at temperatures ranging from −4 °C to 24 °C, which are lower than many materials commonly used in building applications. A sample presented a thermal resistance that is 8%, 10%, and 45% higher than those of rock wool, polyisocyanurate (PIR), and fiberglass, respectively.

## 1. Introduction

The building manufacturing industry is a significant source of greenhouse gas emissions and the consumption of non-renewable resources, accounting for 40% of global energy consumption and 30% of total carbon dioxide emissions [1]. In 2021, Canada committed to achieve carbon neutrality by the end of 2050 [2]. Currently, most building materials are made of unsustainable materials, such as glass wool, fiberglass, rock wool, cement, extruded rock wool, polyurethane (PU), polystyrene (XPS), and expanded polystyrene (EPS) [3]. In spite of their numerous advantages, such as low density, low cost, good mechanical properties, outstanding thermal and acoustic performance, good dimensional stability, and flame resistance, conventional isolating materials produce a significant carbon footprint, contribute to the degradation of our environment, and require a large amount of energy during their manufacturing process [4,5,6,7,8,9,10].

To achieve carbon neutrality by 2050 while retaining equivalent properties to those of unsustainable materials, researchers have considered the use of agricultural waste [11] or natural fibers, including hemp [12], flax [13], kapok [14], bamboo [15], coconut [16], cotton [17], kenaf [18], jute [19], and milkweed [20,21], and demonstrated that these fibers offer excellent thermal and acoustic properties, which make them as effective as conventional materials [20,21].

Given their characteristics, porous nonwovens made from natural fibers and thermoplastics occupy an important place in building applications [22,23,24,25]. Indeed, blending natural fibers with thermoplastic fibers to produce nonwovens is a well-known method to reduce the impact of the inherent defects caused by the morphological irregularities of natural fibers on the properties of nonwovens. For environmental reasons, it is preferable to use a fiber grown close to the manufacturing area. In North America, milkweed is a good candidate for this purpose. Its fiber presents a hollow structure, a large lumen, and a low density [26,27,28] and provides tremendous thermal and acoustic properties [20,21]. Additionally, its cultivation does not require water intake, pesticides, or fertilizers [28].

Our previous studies have shown that nonwovens composed of milkweed, polyester (PET), and polylactic acid (PLA) fibers exhibit excellent acoustic and thermal insulation properties for textile applications [21]. PET and PLA are semicrystalline thermoplastics that offer low density. PET provides thermal and acoustic insulation, while PLA is a sustainable, biodegradable thermoplastic that has been shown to improve dimensional and mechanical stability in natural fiber-reinforced nonwovens, including flax, jute, and kapok [29].

Many studies have been conducted on the thermal properties of milkweed nonwovens produced using an air-laid process for use in textile and automotive applications [20,21,30]. The air-laying process has been shown to exhibit an overall yield rate of 90%, which is significantly higher than the carding process [20]. The thermal conductivity of a nonwoven made of milkweed, polyester, and PLA fibers was evaluated [21]. It was found that its thermal conductivity ranges from 32.55 to 35.32 mW/mK, much less than the limit of 70 mW/mK for thermal insulators [31].

Several authors have studied the acoustic properties of milkweed nonwovens in combination with other fibers such as PET, polypropylene, modal, and cotton using an impedance tube [32,33,34,35]. Hasani et al. studied Estabragh (milkweed)/hollow-polyester nonwovens and found that nonwovens made of pure milkweed exhibit the best sound absorption properties, with a noise absorption coefficient (NAC) close to 0.8 [32]. The authors attributed this result to the diameter of the milkweed lumen, which creates frictional losses. Similarly, Bihola studied punch nonwovens made of Estabragh or kapok in combination with modal fibers and found that the sound absorption increases with milkweed content [35]. They assessed that this is due to a larger number of fibers per unit area [35]. In a recent study, the authors measured the sound absorption coefficient of nonwovens made from milkweed and PLA fibers and found higher values than for jute/polyethylene nonwovens [20]. To the best of the authors’ knowledge, empirical acoustic models have not been tested on milkweed nonwovens. Many empirical models have been developed to predict the behavior of porous absorbers, such as Delany and Bazley, Miki, and Johnson–Champoux–Allard (JCA).

The objective of the present study was to investigate milkweed-based nonwovens as acoustic and thermal insulators for building applications. The acoustic properties were evaluated using an impedance tube and the JCA acoustic model. To the best of the authors’ knowledge, no empirical acoustic model has been used to determine the acoustic properties of this type of material.

## 2. Materials and Methods

### 2.1. Material

**Figure 1:**
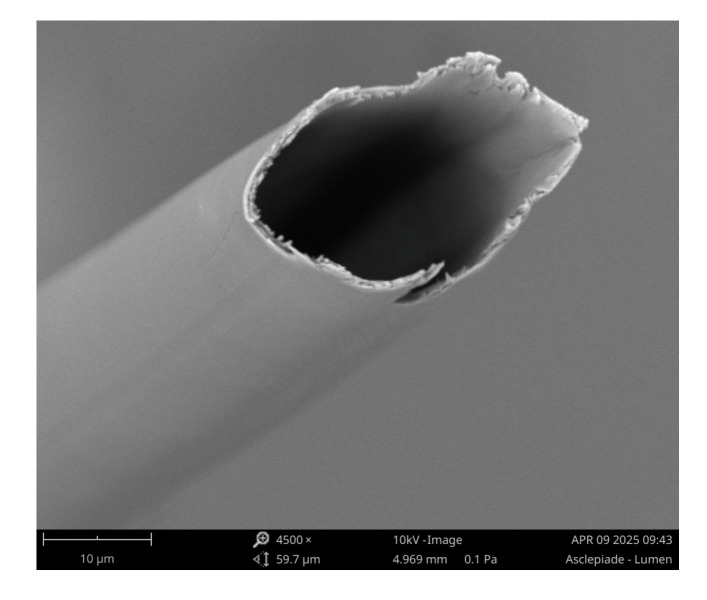
Milkweed fiber at 4500 X magnification.

In our previous study, nonwovens composed of milkweed, PET, and PLA fibers have been produced using the Spike air-laid process, with the goal of producing a thermal insulating material for winter jackets [21]. The thermal insulation fillings had a mass per unit area ranging from 55 to 170 g/m^2^ and a thickness that varied from 4.80 to 10.45 mm. The fiber contents have been modified to evaluate their impact on the functional properties of the nonwovens. The objective of the present work is to develop 25.4 mm thick nonwovens using the same process for building applications. The nonwovens consisted of milkweed, core/sheath fibers (PET core and a polyolefin sheath in a 50:50 ratio) (LM 2.2), and PLA fibers. Figure 1 displays the microstructure of a milkweed fiber obtained by a scanning electron microscope (SEM). A commercially available thermal insulation material made of 100% hemp was purchased from Sprinterfreak (Saint-Roch-de-l’Achigan, QC, Canada) for comparison purposes.

In a previous study, the authors found that the composition of milkweed fibers is 40–45% cellulose, 35–40% hemicellulose, 15% lignin, 3% free sugars, and 3% wax [36].

### 2.2. Sample Preparation

**Figure 2:**
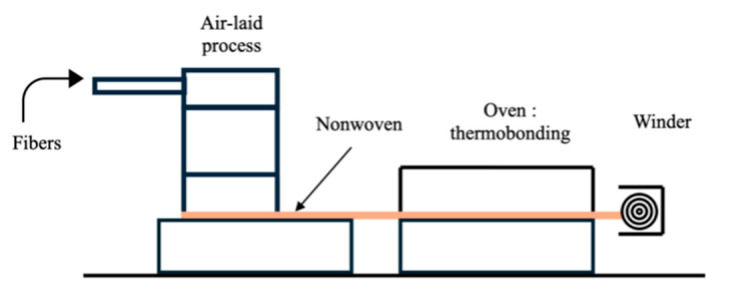
Production line of nonwoven fabrics using an air-laid process [21].

Nonwovens were manufactured using the air-laid Spike process and consolidated through thermal bonding at 150 °C. Figure 2 presents a schematic design illustrating the different steps of the process. More details regarding the fiber properties and the process are available in a previous study [17].

**Figure 3:**
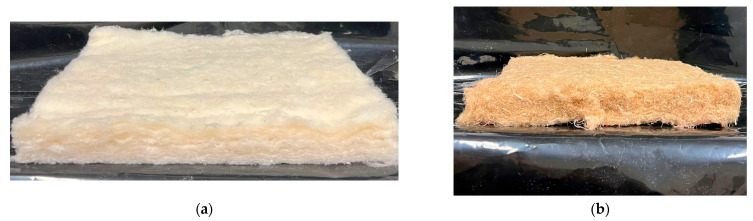
(**a**) Nonwoven; (**b**) the hemp reference.

**Figure 4:**
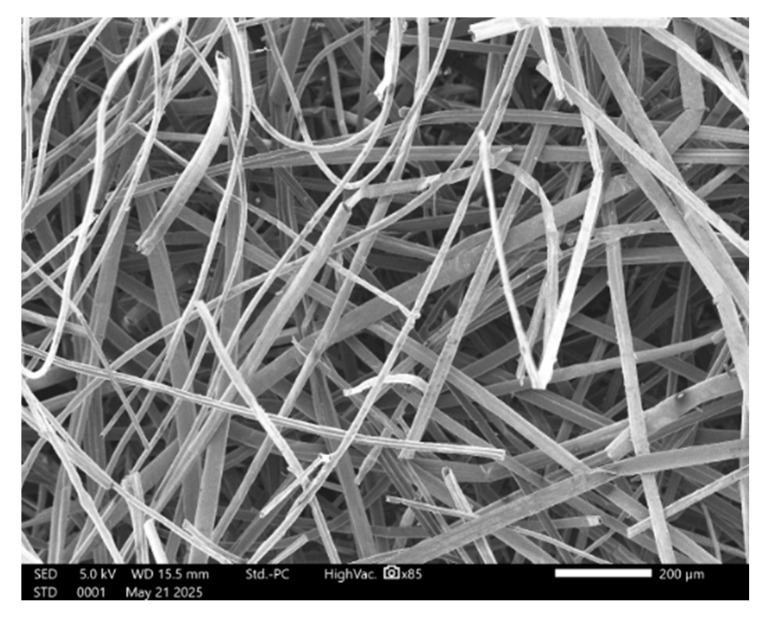
Microstructure of the nonwoven.

Figure 3 shows the nonwoven and hemp reference, whereas Figure 4 presents the internal structure of the nonwoven.

In Figure 4, the homogeneous inner structure of the nonwoven is visible, as well as the fibers that are well-dispersed and randomly distributed.

The composition of the different nonwovens is presented in Table 1 [21].

### 2.3. Physical Properties

#### 2.3.1. Thickness

The thickness (TH) measurement was carried out according to ISO 9073-2 [37], using a pressure of 0.1 kPa. At least ten measurements were obtained per sample.

#### 2.3.2. Bulk Density

The bulk density was measured using an Argon porosity meter (Mecanum Instruments, Sherbrooke, QC, Canada) following the pressure/mass method [38]. At least five measurements were carried out per sample.

### 2.4. Thermal Characterization of Samples

Measurements of thermal conductivity were conducted using a heat-flow meter (FOX 314, TA Instruments, New Castle, DE, USA), following the two-plate method according to ASTM C518 [39]. Different temperatures were tested: 24 °C, 10 °C, 5.5 °C, 0 °C, and −4 °C, with a temperature gradient of around 20 °C. At least five measurements were taken for each nonwoven. The thermal resistance (R) was calculated using Equation (1):

(1) $$R=\;\frac{TH}{\lambda }\cdot 5.678$$

where *R* is the thermal resistance in ($h\cdot {f_{t}}^{2}\cdot {}^{\circ}F$)/Btu, TH the thickness of the sample in m, and λ the thermal conductivity in W/m·K.

### 2.5. Acoustic Characteristics Measurement

#### 2.5.1. Non-Acoustical Parameters

The porosity (ϕ) was measured using a porosity meter (Mecanum Instruments, Sherbrooke, QC, Canada) following the pressure/mass method with argon gas. At least five measurements were recorded for each sample.

Airflow resistivity was conducted with an airflow resistance meter with a sample diameter of 99.8 mm (Mecanum Instruments, Sherbrooke, QC, Canada) according to ASTM C522-03 [40]. At least five measurements were conducted for each sample.

The tortuosity was evaluated with a tortuosity meter by reflection (Mecanum Instruments, Sherbrooke, QC, Canada). At least five measurements were performed for each material.

#### 2.5.2. Measurements of the Sound Absorption Coefficient

**Figure 5:**
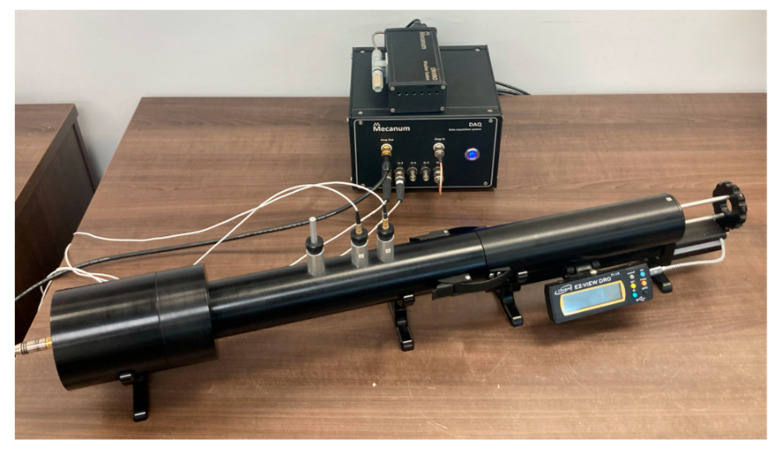
Impedance tube (Mecanum Instruments, Sherbrooke, QC, Canada).

First, the sound absorption coefficient values were measured using an impedance tube (Mecanum Instruments, Sherbrooke, QC, Canada) with an internal diameter of 44 mm according to ASTM E-1050 [41]. For each material, five samples were tested. Figure 5 illustrates the impedance tube used for measuring the sound absorption coefficient.

The materials could be rated based on their random sound incidence coefficient, as described in ISO 11654 [42]. This coefficient is derived from the relationship between the random and normal sound incidence, as described by Wang et al. as follows [43] and shown in Equation (2):

(2) $$f=0.4294\sin\left(-2.7370\alpha \right)-2.2282\alpha^{2}+3.4458\alpha +0.0017$$

where f is the random sound incidence coefficient and α the normal sound incident.

The ISO 11654 categorizes materials based on their weighted sound absorption value ($\alpha_{w}$), which is calculated from the practical sound absorption coefficient ($\alpha_{pi}$), which was obtained from Equation (3):

(3) $$\alpha_{pi}=\frac{\left(\alpha_{i1}+\;\alpha_{i2}+\alpha_{i3}\right)}{3}$$

where, $\alpha_{i1}$, $\alpha_{i2}$, and $\alpha_{i3}$ represent one-third octave band sound absorption coefficients. $\alpha_{pi}$ was calculated for 250, 500, 1000, 2000, and 4000 Hz. $\alpha_{w}$ is the average of the $\alpha_{pi}$ over all octave bands.

Second, the sound absorption coefficient values were determined using an empirical acoustic model. Many models predict the behavior of sound absorption, such as the Delany–Bazley and Miki models, which depend on a single parameter, resistivity. In this study, the Johnson–Champoux–Allard (JCA) model has been chosen for its application to homogeneous materials containing fibers randomly distributed as shown in Figure 4 [44]. Unlike the Delany–Bazley and Miki models, the JCA model considers the internal structure of the materials and depends on five parameters (airflow resistivity, open porosity, tortuosity, viscous characteristic length, and thermal characteristic length).

##### Johnson–Champoux–Allard Model

The aim of modeling the JCA parameters is to simulate the absorption coefficient of the nonwovens at a different thickness from the initial one, for comparison with the reference sample. In our case, the viscous length and thermal length were determined using the inverse method of equivalent fluid for fibrous materials, as implemented by the Foam-X 2024 software; meanwhile, airflow resistivity, open porosity, and tortuosity were measured experimentally. The inverse method is based on the sound absorption coefficient measured using a standardized impedance tube. From the sound absorption coefficient values, the inverse method for an equivalent fluid is applied, and the five-parameter JCA can be determined. The equations, including the five parameters, are presented in Equations (4)–(8) respectively:

(4) $$Z_{s}\left(f\right)=Z_{c}(w)\cdot coth(K_{c}\left(w\right)TH)$$

where $Z_{s}(f)$ is the surface impedance, $Z_{c}\left(w\right)$ is the characteristic impedance, $K_{c}\left(w\right)$ is the propagation constant, and TH is the thickness.

(5) $$\rho \left(w\right)=\alpha_{\propto }\rho_{air}\left[1+\frac{\sigma \phi }{jw\alpha_{\propto }\rho_{air}}{\left(1+\frac{4i\alpha_{\propto }^{2}\eta w\rho_{air}}{{\left(\sigma ⋀\phi \right)}^{2}}\right)}^{0.5}\right]$$

(6) $$K\left(w\right)=kP_{0}{\left(k-\left(k-1\right){\left[1+\frac{8\eta \alpha_{\propto }\phi }{iw{\wedge^{\prime }}^{2}\phi \rho_{air}\alpha_{\propto }N_{pr}}{\left(1+\frac{4i\alpha_{\propto }^{2}\eta N_{pr}w\rho_{air}}{{\left(\sigma {⋀}^{\prime }\phi \right)}^{2}}\right)}^{0.5}\right]}^{-1}\right)}^{-1}$$

(7) $$Z_{c}=\frac{1}{\phi }\sqrt{\rho (w)\cdot K(w)}$$

(8) $$K_{c}\left(w\right)=w\sqrt{\frac{\rho (w)}{K(w)}}$$

where $\rho \left(w\right)$ is the effective density, $K\left(w\right)$ is the bulk modulus,${\;\alpha }_{\propto }$ is the tortuosity, σ is the airflow resistivity, ϕ is the open porosity, ⋀ is the viscous characteristic length, ${⋀}^{\prime }$ is the thermal characteristic length, η is the air viscosity, $\rho_{air}$ is the air density, $N_{pr}$ is the Prandtl number for air, $P_{0}$ is the atmospheric pressure, and k is the adiabatic constant.

The sound absorption coefficient α can be calculated using Equation (9):

(9) $$\alpha =1-\frac{{\left|Z_{s}\left(f\right)-Z_{0}\right|}^{2}}{{\left|Z_{s}\left(f\right)+Z_{0}\right|}^{2}}=1-\frac{{\left|Z_{s}\left(f\right)-\rho_{0}c_{0}\right|}^{2}}{{\left|Z_{s}\left(f\right)+\rho_{0}c_{0}\right|}^{2}}$$

where $c_{0}$ is the speed of sound in the air.

## 3. Results

### 3.1. Properties of the Nonwovens

Table 2 presents the milkweed content, thickness, and density of the nonwovens and the reference sample.

### 3.2. Thermal Insulation

#### 3.2.1. Thermal Conductivity

Thermal conductivity depends on many parameters, such as the internal structure, density, porosity, moisture content, and temperature [45,46,47]. Indeed, an insulation material can behave differently depending on its environment, whether cold or warm [3]. Table 3 presents the variation of thermal conductivity with temperature for nonwovens and the reference.

It can be observed that the thermal conductivity increases from −4 °C to 24 °C for the nonwovens and reference. Consequently, a linear relationship between the mean temperature and thermal conductivity can be found (Table 3). The same behavior was recorded in other studies involving organic and inorganic building materials [48,49,50,51]. The NW 1 sample exhibits slightly higher thermal conductivity than other nonwovens. This is due to its lower milkweed content (30 wt%). For nonwovens containing 50 or 70% milkweed, no significant difference has been observed. Milkweed nonwovens exhibit a lower thermal conductivity than the hemp reference, which presents a thermal conductivity between 42.44 and 49.43 mW/m·K at a density of 36.42 kg/m^3^. In comparison, milkweed nonwovens have a thermal conductivity ranging from 28.37 to 35.32 mW/m·K, corresponding to a density ranging from 10.96 to 19.22 kg/m^3^. Thus, milkweed nonwovens exhibit better thermal insulation compared to hemp, with a thermal conductivity that is 40% to 50% lower.

**Figure 6:**
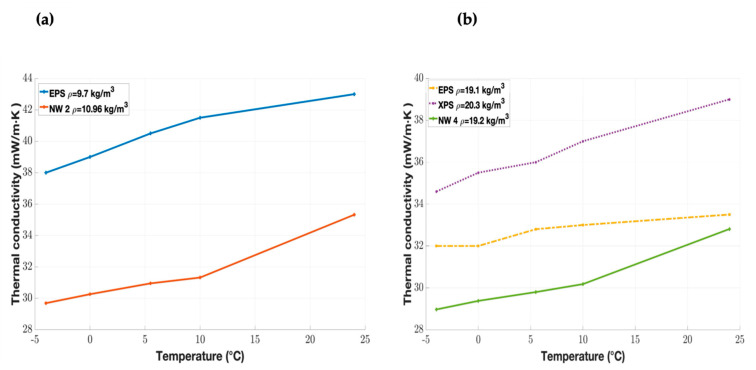
Comparison of the thermal conductivity of milkweed nonwovens and synthetic materials [52] at densities of (**a**) 10.96 kg/m^3^ and (**b**) 19.2 kg/m^3^.

Table 4 and Figure 6 compare the thermal conductivity of the milkweed nonwovens with common organic and inorganic materials used in building applications.

The milkweed materials exhibit superior thermal insulation properties compared to other common inorganic and organic materials (Table 4). In fact, the NW 2 and NW 4 samples provide better thermal performance compared to the EPS and XPS samples for the same density, as they have lower thermal conductivity values [53]. Furthermore, according to DIN 4108, a material is classified as a good insulator if its thermal conductivity is below 70 mW/m·K [31].

To conclude, milkweed nonwovens exhibit superior thermal insulation properties in both cold and warm environments compared to many organic and inorganic materials commonly used in building applications.

#### 3.2.2. Thermal Resistance

Table 5 presents the thermal resistance values of nonwovens and the hemp reference for a thickness of 25.4 mm at different temperatures.

The thermal resistance depends on the thermal conductivity and thickness, and increases as the temperature decreases for all the samples. Like thermal conductivity, nonwovens made of 30 wt% milkweed fibers show a lower thermal resistance than those containing higher milkweed contents. Furthermore, increasing the milkweed content from 50 wt% to 70 wt% does not enhance the thermal resistance, as indicated in Table 2 and Table 5.

**Figure 7:**
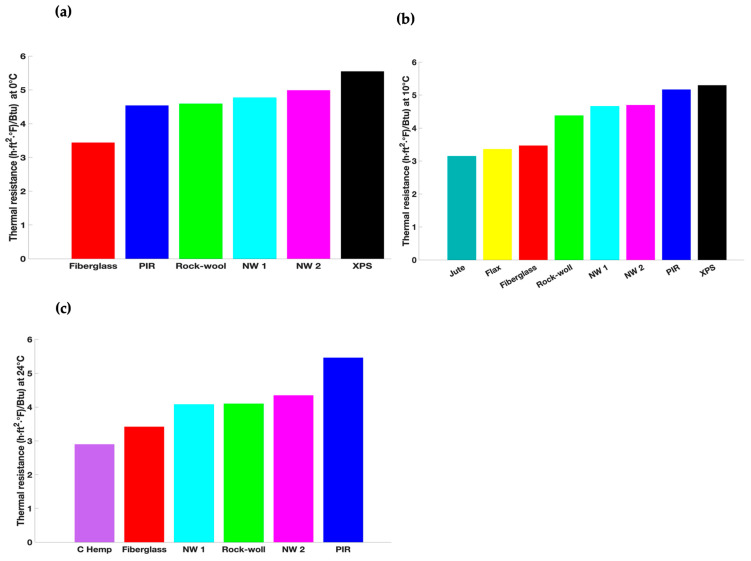
Thermal resistance of NW 1 and NW 2 samples compared to common organic and inorganic materials at (**a**) 0 °C, (**b**) 10 °C, and (**c**) 24 °C.

Figure 7 displays the thermal resistance values of the NW 1 and NW 2 samples and several materials at three temperatures (0, 10, and 24 °C).

The thermal resistance of fiberglass, rock wool, polyisocyanurate (PIR), XPS, jute, flax, and commercial hemp (C hemp) was calculated based on the thermal conductivity reported in the following studies: Ref. [54] for fiberglass, rock wool, PIR, and XPS, Ref. [12] for jute and flax, and Ref. [55] for C hemp.

The NW 2 sample has a thermal resistance value of 4.99 ($h\cdot {f_{t}}^{2}\cdot {}^{\circ}F$)/Btu at a temperature of 0 °C, which is 8, 10, and 45% higher than for rock wool, PIR, and fiberglass, respectively. At 10 °C, this sample provides superior thermal insulation as compared to jute, flax, fiberglass, and rock wool. Based on the results shown in Figure 7, the NW 1 and NW 2 samples can be as thermally efficient as materials made from natural or synthetic fibers.

### 3.3. Acoustic Properties

#### 3.3.1. Analysis of Sound Absorption Coefficient with Impedance Tube

**Figure 8:**
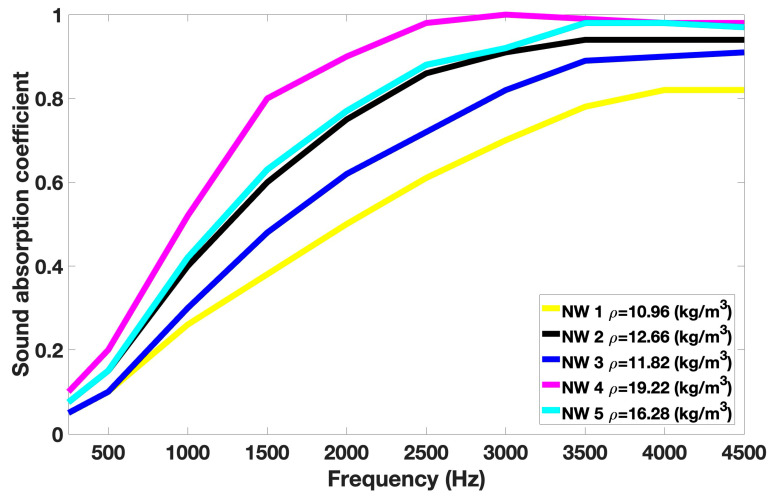
Sound absorption coefficient values versus frequency.

Figure 8 shows the sound absorption coefficient for all nonwovens at frequencies ranging from 250 to 4500 Hz.

The sound absorption coefficient increases with density, resulting in more interaction between the fibers and energy dissipation. The NW1 sample, which has the lowest density (10.96 kg/m^3^), presents the lowest sound absorption properties for frequencies ranging from 250 to 4500 Hz. The NW 2 sample, with a density of 12.66 kg/m^3^, exhibits a better sound absorption coefficient than NW 3 (density of 11.82 kg/m^3^) because of its higher milkweed content (70% versus 50%). Hasani et al. studied the noise absorption coefficient (NAC) of nonwovens made of Estabragh and hollow PET fibers [32]. They showed that a nonwoven containing more Estabragh led to a higher NAC, which would be due to the larger diameter of Estabragh fibers compared to the hollow PET fibers. Xueting Liu et al. studied the sound absorption coefficient of nonwovens made from kapok and hollow PET fibers at different contents [56]. For a density of 14.45 kg/m^3^ and a thickness of 20 mm, they found a sound absorption coefficient ranging from 0.20 to 0.25 and 0.65 to 0.82 for frequencies of 500 Hz and 2500 Hz, respectively. These values are similar to those of NW 5, which has approximately the same density (16.28 kg/m^3^) and thickness (25.4 mm).

#### 3.3.2. Analysis of Sound Absorption Coefficient Using the JCA Model

Table 6 presents the five parameters of the JCA model for the nonwoven samples: airflow resistivity, porosity, tortuosity, viscous characteristic length, and thermal characteristic length.

Airflow resistivity increases with density; this result has been consistently observed in numerous studies [57,58]. All nonwovens are highly porous, exhibiting a porosity of at least 98%. According to Allard et al., for fibrous materials with a low density, which is the case in the present study, the tortuosity is close to 1 [44]. The thermal characteristic length is twice the viscous characteristic length for all samples. Allard et al. indicated that for low-density fibrous materials, the interaction between fibers can be neglected, and consequently, the following relationship can be established: ⋀’ = 2 ⋀.

**Figure 9:**
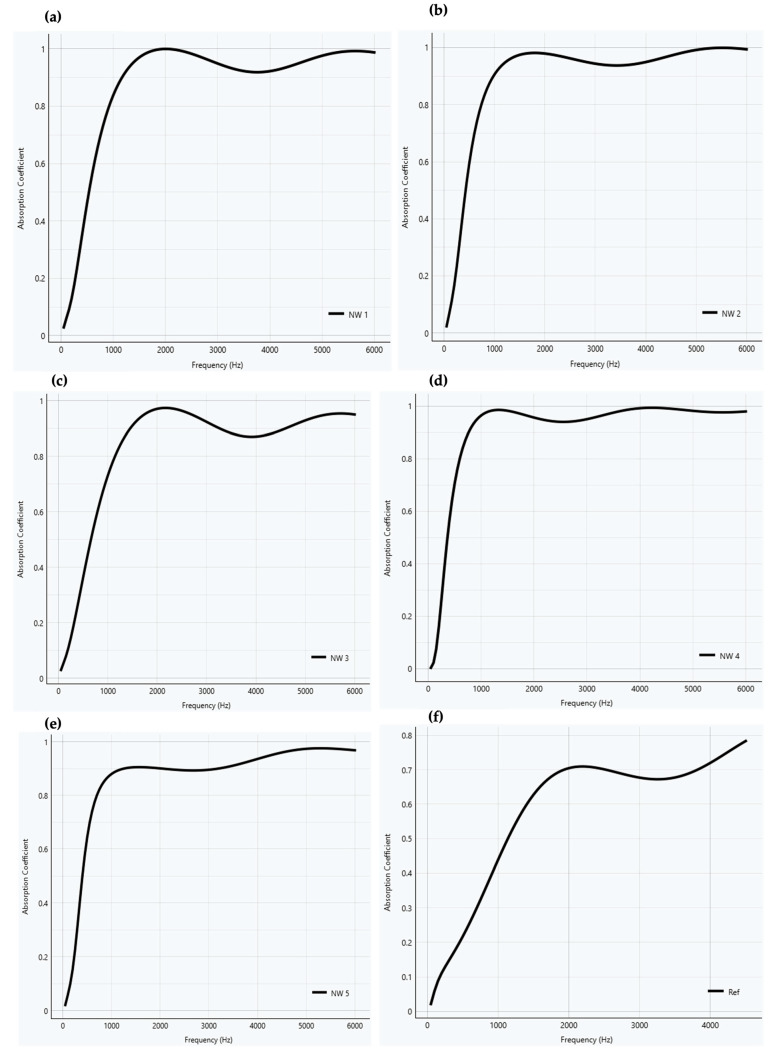
Sound absorption coefficient versus frequency for the nonwoven and reference samples for a thickness of 50 mm: (**a**) NW 1, (**b**) NW 2, (**c**) NW 3, (**d**) NW 4, (**e**) NW 5, (**f**) reference.

Figure 9 illustrates the sound absorption coefficient simulated by the Foam-X software for the nonwoven and reference samples for a thickness of 50 mm, which allows a good comparison between the materials.

The sound absorption coefficient of the hemp reference at 2000 and 4500 Hz is equal to 0.70 and 0.78, respectively. By contrast, for all nonwovens, this value is higher than 0.9 and 0.95, respectively. It may be concluded that the nonwovens offer a better acoustic insulation than hemp.

#### 3.3.3. Classification of Absorbing Materials According to ISO 11654

Table 7 presents the acoustic classification of the nonwovens according to ISO 11654 [42].

According to ISO 11654 [42], both the nonwovens and the reference sample can be classified as Class C (highly absorbing), which denotes a material capable of absorbing at least 60% of the sound incident. However, the nonwovens are half the thickness of the reference. Comparing the rating class for all samples with a thickness of 50 mm, nonwovens are classified as classes A or B, indicating they are considered extremely absorbent, with at least 90% and 80% of the sound, respectively.

## 4. Conclusions

This study demonstrates that milkweed nonwovens offer high thermal and acoustic properties for building applications. From the data obtained for thermal conductivity and resistance, and the sound absorption coefficient, the following conclusions may be drawn:

All of the nonwovens exhibit a thermal conductivity below 35 mW/m·K at temperatures ranging from −4 to 24 °C and are ranked as good insulators according to DIN 4108. For the same density range, milkweed nonwovens exhibited thermal conductivity values ranging from 28.37 to 35.32 mW/m·K, which are lower than those for sheep wool (38–54 mW/m·K). Furthermore, milkweed nonwovens exhibit superior thermal resistance values in both cold and warm environments compared to many organic and inorganic materials commonly used in building applications, such as rock wool, PIR, fiberglass, and hemp.

An increase in milkweed content or density leads to higher sound absorption. The simulation of sound absorption using the JCA model has shown that milkweed nonwovens are better sound absorbers than hemp. According to ISO 11654, milkweed nonwovens with a thickness of 25.4 mm can be categorized as Class C absorbers, while increasing the thickness to 50 mm allows them to be classified as Class A or B.

Further properties, such as the vapor permeance, compressive strength, thermal degradation, and fire resistance, must be evaluated to confirm whether milkweed nonwovens could be used as thermal and acoustic materials for building applications.

## Figures and Tables

**Table 1:** Composition of nonwoven sample production.

Samples	MW (wt%)	LM 2.2 (wt%)	PLA (wt%)
1	30	10	60
2	70	10	20
3	50	20	30
4	50	10	40
5	50	40	10

**Table 2:** Characteristics of nonwoven and reference samples.

Sample	MW (wt%)	Thickness (mm)	Standard Deviation (mm)	Density (kg/m^3^)
NW 1	30	25.54	0.16	10.96
NW 2	70	25.53	0.35	12.66
NW 3	50	25.50	0.24	11.82
NW 4	50	25.52	0.46	19.22
NW 5	50	25.51	0.60	16.28
Ref	0	50.02	0.39	33.24

**Table 3:** Thermal conductivity values for all nonwoven samples and for the reference at different temperatures.

Sample	*λ* (mW/m·K) *T* = −4 °C	*λ* (mW/m·K) *T* = 0 °C	λ (mW/m·K) *T* = 5.5 °C	*λ* (mW/m·K) *T* = 10 °C	*λ* (mW/m·K) *T* = 24 °C	Relationship
NW 1	29.69 (0.01)	30.26 (0.05)	30.95 (0.05)	31.32 (0.01)	35.32 (0.04)	*λ* = 0.1995T + 30.091
NW 2	28.37 (0.05)	28.92 (0.01)	29.02 (0.01)	30.34 (0.04)	33.13 (0.03)	*λ* = 0.1727T + 28.729
NW 3	29.55 (0.06)	29.97 (0.02)	30.48 (0.02)	31.32 (0.03)	33.96 (0.09)	*λ* = 0.1593T + 29.905
NW 4	28.97 (0.02)	29.38 (0.01)	29.8 (0.01)	30.18 (0.02)	32.81 (0.02)	*λ* = 0.1365T + 29.259
NW 5	29.58 (0.03)	30.11(0.01)	30.65 (0.02)	31.1 (0.05)	33.65 (0.02)	*λ* = 0.1440T + 29.995
Ref	42.44 (0.12)	43.4 (0.12)	44.05 (0.14)	44.67 (0.15)	49.43 (0.14)	*λ* = 0.2450T + 43.058

**Table 4:** Thermal conductivity of common organic and inorganic materials used in building applications.

Material	Density (kg/m^3^)	λ (mW/m·K)	Reference
Milkweed nonwoven	10.96–19.22	28.37–35.32	[21]
Blanket Fiberglass	12–56	33–40	[45]
Blanket Polyethylene	35–40	41	[45]
Rockwool Loose-fill Blown-in Poured-in	24–36	46–54	[45]
Kenaf	30–180	34–43	[52]
Sheep wool	10–25	38–54	[52]

**Table 5:** Thermal resistance values of nonwovens and the hemp reference for a thickness of 25.4 mm at different temperatures.

Sample	*R* $(h\cdot {f_{t}}^{2}\cdot {}^{\circ}F$)/Btu *T* = −4 °C	*R* $(h\cdot {f_{t}}^{2}\cdot {}^{\circ}F$)/Btu *T* = 0 °C	*R* $(h\cdot {f_{t}}^{2}\cdot {}^{\circ}F$)/Btu *T* = 5.5 °C	*R* $(h\cdot {f_{t}}^{2}\cdot {}^{\circ}F$)/Btu *T* = 10 °C	*R* $(h\cdot {f_{t}}^{2}\cdot {}^{\circ}F$)/Btu *T* = 24 °C
NW 1	4.86	4.77	4.66	4.60	4.08
NW 2	5.08	4.99	4.87	4.75	4.35
NW 3	4.88	4.81	4.73	4.60	4.25
NW 4	4.98	4.91	4.84	4.78	4.4
NW 5	4.88	4.79	4.7	4.64	4.29
Ref	3.40	3.32	3.27	3.23	2.92

**Table 6:** Physical and non-acoustical properties of the nonwovens.

Samples	Airflow Resistivity σ (Pa·s/m^2^)	Porosity ε	Tortuosity $\alpha_{\propto }$	Viscous Characteristic Length ⋀ (μm)	Thermal Characteristic Length ${⋀}^{\prime }$ (μm)
NW 1	9986	0.982	1.01	296.5	592.9
NW 2	14,177	0.985	1.03	220.2	440.4
NW 3	12,493	0.982	1.02	347.1	694.2
NW 4	21,150	0.997	1.05	55.6	111.2
NW 5	20,370	0.997	1.05	350.2	700.5

**Table 7:** Acoustic classification of absorbing materials according to ISO 11654.

Sample	$\alpha_{w}$ Thickness 25 mm	Rating Class Thickness 25 mm	$\alpha_{w}$ Thickness 50 mm	Rating Class Thickness 50 mm
NW 1	0.6	C	0.8	B
NW 2	0.7	C	0.85	B
NW 3	0.6	C	0.85	B
NW 4	0.7	C	0.9	A
NW 5	0.7	C	0.9	A
Ref			0.7	C

## Data Availability

The original contributions presented in this study are included in the article/supplementary material. Further inquiries can be directed to the corresponding author(s).
